# Aberrant methylation and silencing of *IRF8* expression in non-small cell lung cancer

**DOI:** 10.3892/ol.2014.2234

**Published:** 2014-06-11

**Authors:** MAKOTO SUZUKI, KOEI IKEDA, KENJI SHIRAISHI, AYAMI EGUCHI, TAKESHI MORI, KENTARO YOSHIMOTO, HIDEKATSU SHIBATA, TAKAAKI ITO, YOSHIFUMI BABA, HIDEO BABA

**Affiliations:** 1 Department of Thoracic Surgery, Graduate School of Medical Sciences, Kumamoto University, Kumamoto 860-8556, Japan; 2 Department of Pathology and Experimental Medicine, Graduate School of Medical Sciences, Kumamoto University, Kumamoto 860-8556, Japan; 3 Department of Gastroenterological Surgery, Graduate School of Medical Sciences, Kumamoto University, Kumamoto 860-8556, Japan

**Keywords:** *IRF8*, pyrosequencing, methylation, expression

## Abstract

The aim of the present study was to investigate the aberrant methylation and altered expression of the interferon regulatory factor 8 (*IRF8*) gene in non-small cell lung cancer (NSCLC). Pyrosequencing assays were performed on 191 tumor specimens from NSCLC patients. The changes in *IRF8* mRNA expression, prior to and following treatment with a demethylating agent and methylation itself, were examined in 13 lung cancer cell lines by quantitative polymerase chain reaction (qPCR) and pyrosequencing. IRF8 protein expression was examined in 94 of the 191 NSCLC specimens by immunohistochemical analysis. The *IRF8* methylation level was significantly higher in the tumor tissues than in matched non-malignant lung tissues (P<0.0001). *IRF8* was more frequently methylated in tumor tissues compared with matched non-malignant lung tissues, as defined by a predetermined cut-off value (P<0.0001). The *IRF8* methylation level was strongly correlated with the change in mRNA expression in lung cancer cell lines and with the protein expression level in primary tumors. The *IRF8* gene was more frequently methylated in patients without an epidermal growth factor receptor (EGFR) mutation than in patients with an EGFR mutation (P=0.015). *IRF8* methylation correlated with recurrent prognosis in adenocarcinomas (log-rank test, P=0.048). IRF8 protein expression was frequently silenced in males, smokers, patients with non-adenocarcinoma or with wild-type EGFR, or in an advanced stage. *IRF8* is often silenced by its methylation, which is a frequent event in NSCLC and, therefore, methylation of *IRF8* may act as a prognostic marker for recurrence. Analysis of *IRF8* methylation status may provide novel opportunities for improved prognosis and therapy of resected NSCLC.

## Introduction

Lung cancer is widely prevalent and is one of the leading causes of cancer-related mortality worldwide. Despite recent advances in the molecular analysis and understanding of lung cancer, the associated mortality rate has not changed significantly in the last 30 years (1).

The silencing of tumor suppressor genes is frequently caused by epigenetic changes rather than mutations. These changes are important in lung cancer, as aberrant methylation of tumor suppressor genes is considered to be one of the contributing factors to the carcinogenesis of non-small cell lung cancer (NSCLC) (2).

Interferon regulatory factor 8 (IRF8), also known as interferon consensus sequence-binding protein, is a transcription factor belonging to the interferon regulatory factor family. It is induced by interferon gamma (3) and is an important regulator of immunity and other physiological processes, including oncogenesis (4). In the immune system, IRF8 is essential for macrophage, dendritic cell and B-cell development and function (5). Irf8^−/−^ mice develop a chronic myelogenous leukemia (CML)-like syndrome (6). IRF8 is absent from CML patients (7,8) and therapeutic interferon (IFN)-alpha for CML induces *IFR8* expression *in vivo* (9). The tumor suppressor activity of IRF8 has been observed in non-hematopoietic tumors. Following induction by IFN-γ, caspase-1 and IRF8 sensitize human colon carcinoma cells to Fas-mediated apoptosis (10). There have been studies demonstrating aberrant methylation of the *IRF8* gene in solid tumors. Furthermore, repression of *IRF8* by aberrant methylation is a molecular determinant of apoptotic resistance and the metastatic phenotype in human metastatic colon carcinoma cell lines and murine mammary carcinoma with lung metastasis (3). Also, aberrant methylation of the *IRF8* gene in nasopharyngeal, esophageal and multiple other carcinomas has been reported (11).

Considering the previous methylation studies of IRF8 in cancer sites, including lung cancer cell lines (11), we hypothesized that *IRF8* may also be frequently methylated in NSCLC. In the present study, we examined *IRF8* methylation and mRNA expression in lung cancer cell lines, and *IRF8* methylation and protein expression in primary NSCLCs, and compared the results with the clinicopathological features.

## Materials and methods

### Patients and cell lines

Specimens were obtained from 191 consecutive patients who underwent thoracic surgery at Kumamoto University (Kumamoto, Japan) from 2010 to 2012. None of these patients underwent pre-operative chemotherapy, pre-operative radiotherapy or chemoradiotherapy. Informed consent was obtained from each patient. The study design was approved by the ethics review board of the Kumamoto University and patients provided written informed consent.

NSCLC cell lines (11 in total; PC4, PC10, LU99c, HCC15, H63, H157, H460, HUT15, HUT29, HUT70 and A549) and two SCLC cell lines (SBC-1 and SBC-5) were used in this study. PC4, PC10, HUT15, HUT29 and HUT70 were self-established (12), while LU99c, A549, SBC-1 and SBC-5 were purchased from the Japanese Collection of Research Bioresources Cell Bank (Osaka, Japan) and HCC15, H63, H157, and H460 were generously donated by Dr. Gazdar of the University of Texas Southwestern Medical Center (Dallas, TX, USA).

### Treatment with 5-aza-2′-deoxycytidine (5-Aza-CdR)

Tumor cell lines were incubated in culture medium with 1 μM of the demethylating agent 5-Aza-CdR (Sigma-Aldrich, St. Louis, MO, USA) for 6 days, with medium changes on days 1, 3 and 5. Cells were harvested and RNA was extracted on day 6, as described previously (13).

### Reverse transcription-polymerase chain reaction (RT-PCR)

An RT-PCR assay was used to examine mRNA expression. Total RNA was extracted from samples with TRIzol (Invitrogen Life Technologies, Carlsbad, CA, USA) following the manufacturer’s instructions. The RT reaction was performed on 4 μg of total RNA using deoxyribonuclease I and the SuperScript II First-Strand Synthesis system with the oligo (dT) primer System (Invitrogen Life Technologies). Aliquots of the reaction mixture were subsequently used for PCR amplification. Quantitative PCR (qPCR) was performed using SYBR Premix Ex Taq (Perfect Real Time; Takara Bio, Inc., Shiga, Japan) and Thermal Cycler Dice^®^ Real Time System TP850 software. The results were analyzed using the comparative Ct method (ΔΔCt) to compare the relative expression of each target gene prior to and following 5-aza-CdR treatment according to the user manual, and the ratio (5-aza-CdR/mRNA) was obtained. GAPDH was co-amplified with the target genes and served as an internal standard. Primer sequences were identical to those of the endogenous human target genes as confirmed by a BLAST search. For all RNA examined, the GenBank accession numbers are listed in parentheses. The qPCR primer sequences were as follows: *IRF8* (NM_002163.2) forward primer, TCCGGATCCCTTGGAAACAC and reverse primer, CCTCAGGAACAATTCGGTAA; GAPDH (NM_002046.3) forward primer, TGAACGGGAAGCTCACTGG and reverse primer, TCCACCACCCTGTTGCTGTA. Genomic DNA was not amplified with these primers, as all sequences were generated from cDNA.

DNA was treated with sodium bisulfite using EpiTect Bisulfite kits according to the manufacturer’s instructions (Qiagen Inc., Valencia, CA, USA). Subsequent PCR and pyrosequencing for each gene was performed using the PyroMark kit (Qiagen Inc.) as described previously (14,15). The PCR conditions were as follows: 45 cycles of 95°C for 20 sec, 50°C for 20 sec and 72°C for 20 sec, followed by 72°C for 5 min. The biotinylated PCR products were purified and denatured prior to pyrosequencing with the Pyrosequencing Vacuum Prep Tool (Qiagen Inc.) in the PyroMark Q96 MD system (Qiagen Inc.). The nucleotide dispensation order was as follows: YGYGATTGAAATAGGAGTATYGAABGTG. The amount of C relative to the sum of the amounts of C and T at each CpG site was calculated as a percentage (i.e., 0–100%). The average of the relative amounts of C in the CpG sites was used as the overall methylation level of each gene in a given tumor.

Detection of epidermal growth factor receptor (*EGFR*) gene mutations was performed using the Cycleave method as described previously (16).

### Immunohistochemistry

Three serial 5-μm sections obtained from 94 formalin-fixed, paraffin-embedded lung cancer samples were stained either with standard hematoxylin and eosin or with the biotin streptavidin-peroxidase method. IRF8 protein expression was determined using mouse monoclonal antibody (ab61750; Abcam, Tokyo, Japan) diluted to 1 μg/μl. The primary antibody was incubated overnight at room temperature. Tumor cells with IRF8 cytoplasmic and/or membrane immunohistochemical expression were considered positive for expression. Staining was assessed using three semi-quantitative categories based on the percentage of stained (positive) tumor cells; absence of staining or <10% positive cells (low), 10–50% positive cells (moderate) and >50% positive cells (high). Cases were considered positive when >10% of the tumor cells demonstrated cytoplasmic and/or membrane expression.

### Statistical analysis

The Fisher’s exact test and Mann-Whitney U test were applied to assess the association between categorical variables. P<0.05 was considered to indicate a statistically significant difference. All P-values were two-sided. To determine the appropriate methylation cut-off value, each methylation level was subdivided into two cohorts using receiver operating characteristic (ROC) curve analysis (17). All statistical analyses were performed using SPSS 16.0 for Windows (SPSS, Inc., Chicago, IL, USA).

## Results

### Aberrant methylation of IRF8 in primary tumors

**Figure 1:**
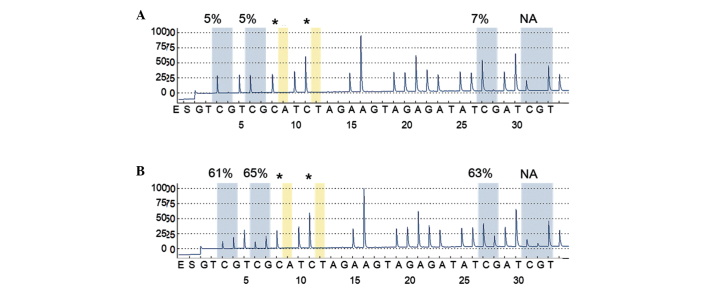
Representative examples of measurement of *IRF8* methylation level using pyrosequencing. (A) Unmethylated *IRF8* non-malignant lung tissue (methylation level, 6%). (B) Methylated *IRF8* tumor (methylation level, 63%). The percentages are the proportion of C at each CpG site following bisulfite conversion, and the methylation level of each CpG site is estimated by the proportion of C (%). An overall *IRF8* methylation level is calculated as the average of the proportion of C (%) at the three CpG sites. ^*^Indicates no residual C at the non-CpG site, ensuring complete bisulfite conversion. *IRF8*, interferon regulatory factor 8; NA, not analyzed.

We examined the methylation level of *IRF8* in 191 NSCLC tissues and matched non-malignant lung tissues, with representative cases illustrated in Fig. 1. Tumor tissues demonstrated significantly higher levels of *IRF8* methylation (mean ± standard deviation, 11.6±10.3%) than matched non-malignant lung tissues (6.5±1.4%; Wilcoxon signed-rank test, P<0.0001). The data indicated that aberrant methylation was a tumor-specific event in NSCLC.

ROC curve analyses were conducted to obtain a cut-off value for *IRF8* methylation. The cut-off value was determined to be 8%, and the frequencies of aberrant methylation in tumors were 45% (85/191) and 8% (16/191) in matched non-malignant lung tissues. Comparisons of tumor tissues with matched non-malignant lung tissues indicated that aberrant methylation of *IRF8* gene was a tumor-specific event (Fisher’s exact probability test; P<0.0001), despite the fact that tumor tissues consisted of mixtures of tumor cells and non-malignant cells.

Following this, the clinicopathological features were compared with the frequencies of aberrant methylation of *IRF8* in NSCLC (Table I). There were no significant associations with age, gender, smoking history, stages or histological types. The methylation frequency of the *IRF8* gene was significantly higher in patients without *EGFR* mutations compared with patients with *EGFR* mutations (P=0.015).

**Figure 2:**
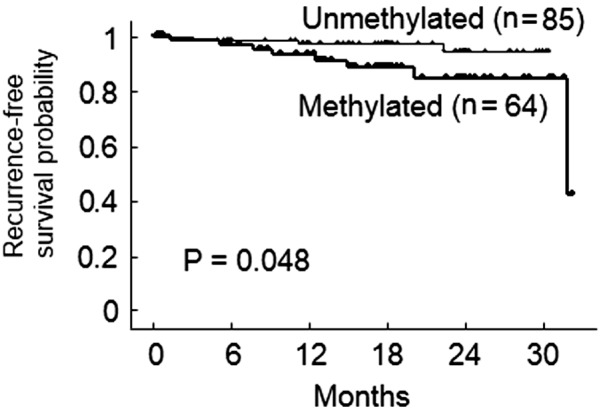
Kaplan-Meier curves for recurrence-free survival of patients with adenocarcinoma. The 2-year recurrence-free survival rates were 94.6% for patients with unmethylated *IRF8* and 84.3% for patients with methylated *IRF8*. IRF8, interferon regulatory factor 8.

Due to the limited duration of the follow-up period, recurrence-free survival was examined. Of 149 patients with adenocarcinoma, only 11 patients had recurrent disease during follow-up. According to a Kaplan-Meier analysis, the 2-year recurrence-free survival rates of patients with adenocarcinoma were 84.3% in methylated cases and 94.6% in non-methylated cases (Fig. 2; log-rank test, P=0.048). Multivariable analysis was not conducted, as the number of uncensored cases was too small (n=11).

### Correlation between methylation and expression of IRF8 in cell lines and in primary tumors

The methylation level of *IRF8* in the cell lines was examined. According to the cut-off value obtained from primary tumor sample analysis, *IRF8* hypermethylation was present in 10/13 cell lines (Table II). Following this, the expression of *IRF8* mRNA was examined by qPCR prior to and following demethylation with 5-aza-CdR treatment. By calculating the ratios of gene expression prior to and after the treatments, it was determined that IRF8 expression was higher following 5-aza-CdR treatment in 12/13 cell lines (Table II). Hypermethylation and re-expression were observed in the nine cell lines. No re-expression, despite hypermethylation, was observed in one cell line and re-expression was present in three cell lines without hypermethylation. Therefore, the concordance between the change of *IRF8* expression and methylation status was 69% (9/13).

**Figure 3:**
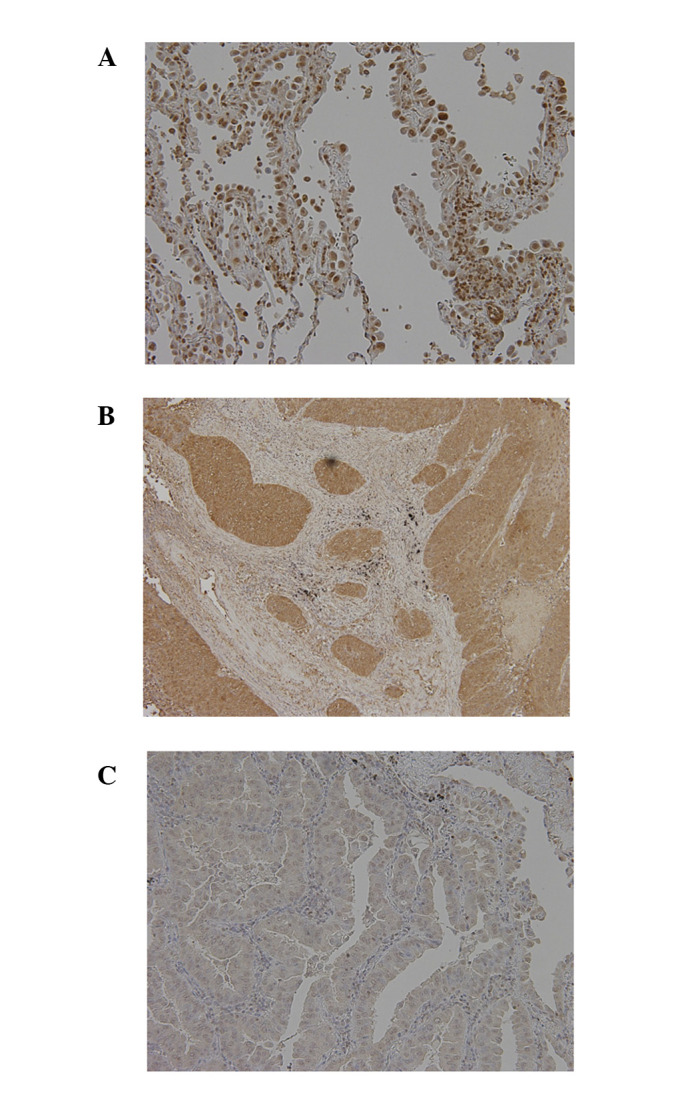
Immunohistochemical staining patterns for IRF8 in resected NSCLCs. (A) An unmethylated *IRF8* adenocarcinoma with a high score (cytoplasmic and/or nuclear immunostaining in >50% of tumor cells; strong staining intensity). (B) An unmethylated *IRF8* squamous cell carcinoma with a high score (cytoplasmic and/or nuclear immunostaining in >50% of tumor cells; strong staining intensity). (C) A methylated *IRF8* adenocarcinoma with a low score (cytoplasmic and/or nuclear immunostaining in <10% of tumor cells; weak staining intensity). *IRF8*, interferon regulatory factor 8; NSCLC, non-small cell lung cancer.

Typical immunostaining patterns for IRF8 in NSCLC are presented in Fig. 3. Using the criteria described in Materials and methods, negative expression of IRF8 was present in 57/94 tumors (61%). *IRF8* hypermethylation occurred in 31/57 tumors lacking expression and hypermethylation was absent from 26/37 tumors expressing IRF8. Thus, a high concordance (61%; 57/94) was observed between hypermethylation and loss of expression in primary tumors (P=0.02).

### Expression of IRF8 in primary tumors

As summarized in Table I, IRF8 protein expression was more frequently downregulated in male than in female patients, in smokers than in non-smokers, in non-adenocarcinomas than in adenocarcinomas, in stage IB-III than in stage IA, and in patients with wild-type *EGFR* than in those with mutated *EGFR*. Of the 94 patients, seven patients had recurrence of disease. One patient exhibited a positive expression of IRF8 while others had no expression of IRF8. The 2-year recurrence-free survival rate for patients with negative expression was 87.9% and with positive expression was 97.1% (log-rank test, P=0.14).

## Discussion

In the present study, it was demonstrated that the re-expression of *IRF8* mRNA occurs in lung cancer cell lines following treatment with a demethylating agent, and that it is correlated with the methylation status of the gene, although the level of re-expression was varied. IRF8 protein expression is also correlated with the methylation status of the gene in primary tumors. These results indicate that methylation was the likely mechanism by which *IRF8* mRNA and IRF8 protein expression was suppressed. There are other possible mechanisms for the downregulation of *IRF8* gene expression, including histone acetylation, loss of heterozygosity or miRNA, or toxicity of the demethylating agent. However, the concordance between methylation and the loss of gene and subsequent protein expression robustly supports the importance of DNA methylation. Previously, adding to the above inactivation mechanisms, mutation of *IRF8* has been reported in patients with disseminated infection caused by bacilli Calmette-Guérin vaccines (18). In the present study, *IRF8* was examined for mutations in 20 NSCLC and non-malignant lung tissue samples; however, we were unable to identify the presence of any (data not shown).

Aberrant methylation of *IRF8* has been reported in multiple carcinoma cell lines, including four lung cancer cell lines (A549, H292, H358 and H1975) by methylation-specific PCR assay (11). These authors also examined the expression of *IRF8* by RT-PCR and observed ectopic *IRF8* expression in nasopharyngeal, esophageal and colon cancer cell lines. *IRF8* expression appeared to suppress colony formation, leading to the conclusion that *IRF8* may act as a tumor suppressor. In the present study, *IRF8* expression and methylation in A549 was examined, and it was identified that the *IRF8* gene was highly methylated and that expression was restored following treatment with a demethylating agent, as determined by a quantitative assay. In another study, it was revealed that exogenous expression of *IRF8* in a metastatic colon cell line restored, at least partially, the sensitivity of the tumor cells to Fas-mediated apoptosis (3). The present study results demonstrated that the *IRF8* gene is highly and frequently methylated in NSCLC in a tumor-specific manner. Furthermore, the data indicated that IRF8 may have an important role in cancer pathogenesis. Further studies are required to understand the function of *IRF8* in lung cancer pathogenesis.

The clinical relevance of *IRF8* methylation in NSCLC has not been reported previously. First, we identified that *IRF8* methylation was significantly more frequent in tumors without an EGFR mutation than in those with an EGFR mutation. Previously, we reported that the myelin and lymphocyte protein (MAL) gene was exclusively methylated with an EGFR mutation (15). It is possible that methylated-type NSCLC and mutant EGFR NSCLC possess different etiologies. Secondly, although based on a relatively small sample size and short-term follow up, *IRF8* methylation was correlated with poorer recurrence-free survival in adenocarcinoma cases. *IRF8* methylation may be clinically useful, utilized as an adjuvant therapy or as a recurrence-prediction marker of adenocarcinoma. Large-scale studies that include longer follow-up periods, may clarify the significance of *IRF8* methylation in NSCLC.

The expression of the IRF8 protein in this setting has not been reported previously. In the present study, it was identified that the expression of IRF8 was frequently silenced in NSCLC cells. Also, the protein was silenced frequently in males, smokers, non-adenocarcinomas and in EGFR wild-type patients. Furthermore, the protein was silenced frequently in advanced stages and the patients with negative protein expression demonstrated recurrence of disease. These data require further investigation, on a larger scale and with longer follow up periods, to elucidate the clinical role of IRF8 protein expression in NSCLC.

In conclusion, the data regarding *IRF8* methylation and expression may provide new insights into NSCLC pathogenesis and therefore have clinical value as potential prognostic indicators.

## Figures and Tables

**Table I:** Methylation (n=191) and protein expression (n=94) of IRF8 in NSCLC patients.

Clinical characteristics of primary tumors (n^a^/n^b^)	Methylated, n (%)	P-value	Negatively stained, n (%)	P-value
Gender				
Male (113/59)	48 (42)	NS	42 (71)	0.009
Female (78/35)	37 (47)		15 (43)	
Age^c^ (years)				
<69/<70 (94/49)	43 (46)	NS	30 (61)	NS
≥69/≥70 (97/45)	42 (43)		27 (60)	
Smoking				
Smoker (110/58)	50 (45)	NS	42 (72)	0.005
Never (81/36)	35 (43)		15 (42)	
Histology				
Adenocarcinoma (149/71)	64 (43)	NS	37 (52)	0.003^d^
Squamous cell carcinoma (28/14)	16 (57)		12 (86)	
Others (14/9)	5 (36)		8 (89)	
Primary tumor stage				
IA (108/52)	51 (47)	NS	26 (50)	0.02
IB-III (83/42)	34 (41)		31 (74)	
EGFR status (n=159/n=73)				
Wild-type (94/45)	51 (54)	0.015	31 (69)	0.049
Mutated type (65/28)	22 (34)		12 (43)	

n^a^, Number of cases examined by pyrosequencing; n^b^, number of cases examined by immunohistochemistry;

c divided into 2 groups by median age (n=191 and n=94, respectively);

d P-value as determined by Fisher’s exact test, compared with non-adenocarcinoma and adenocarcinoma.

IRF8, interferon regulatory factor 8; NSCLC, non-small cell lung cancer; NS, not significant.

**Table II:** *IRF8* methylation and the changes in *IRF8* mRNA expression following treatment with 5-Aza-CdR.

Cell line	Type	Methylation level (%)	5-Aza-CdR/mRNA ratio
SBC-1	SCLC	91.0	0.4
SBC-5	SCLC	97.7	28711
PC14	NSCLC	43.9	24.4
LU99c	NSCLC	66.9	1.6
HCC15	NSCLC	7.3	4.1
H63	NSCLC	5.4	2.3
H157	NSCLC	47.1	3.9
H460	NSCLC	51.2	132
HUT15	NSCLC	6.3	1.6
HUT29	NSCLC	47.1	1.3
HUT70	NSCLC	97.9	61.8
PC10	NSCLC	65.6	7.3
A549	NSCLC	48.4	1.1

*IRF8*, interferon regulatory factor 8; NSCLC, non-small cell lung cancer; 5-Aza-CdR, 5-aza-2′-deoxycytidine; SCLC, small cell lung cancer.

## Abbreviations

*IRF8*: interferon regulatory factor 8
NSCLC: non-small cell lung cancer
